# How can a community of practice support healthcare professionals navigating new roles? a case study of genetic counsellors employed to work in medical specialities

**DOI:** 10.1186/s12913-025-12440-2

**Published:** 2025-02-25

**Authors:** Trang Thu Do, Alison McEwen, Melissa Martyn, Clara Gaff, Belinda Dawson-McClaren

**Affiliations:** 1 Murdoch Children’s Research Institute, Parkville, VIC Australia; 2 Department of Paediatrics, University of Melbourne, Parkville, VIC Australia; 3 Graduate School of Health, University of Technology Sydney, Sydney, Australia

**Keywords:** Communities of practice, Learning platform, Knowledge translation, Genomics, Genetic counsellors

## Abstract

**Background:**

Communities of Practice (CoPs) have been implemented in healthcare settings to enhance knowledge translation and facilitate the implementation of new practices. However, their role in supporting healthcare professionals transitioning to new environments remains under-researched. This study examines a CoP designed for genetic health professionals in Australia who were employed to support the integration of genomics in medical specialities. Informed by the i-PARIHS framework, we explore how the facilitation of a CoP external to the implementation setting can support health professionals implementing innovative practices.

**Methods:**

Data was collected through qualitative interviews with 14 genetic counsellors participating in the CoP through different stages of their new roles, 35 discussion and reflection logs, and workshop and meeting notes. Thematic analyses were carried out to capture the patterns and process of facilitation performed by this CoP, resulting in five overarching themes.

**Results:**

Participants highlighted the unique role of the CoP in forging peer connection and providing emotional support in new environments with a high degree of uncertainty and limited peer support. Through CoP sessions and associated professional development workshops, they benefited from ongoing knowledge acquisition about good practices and innovations. The CoP served as an effective space for identifying and solving problems collectively or escalating emergent issues. Additionally, the CoP helped participants build inter-personal skills to overcome relational challenges and improved communication with non-genetic colleagues about genomics. Critical reflection emerged as both a practice and an impact of the CoP, enabling participants to redefine their roles and adopt future-oriented thinking for the genetic counselling profession.

**Conclusion:**

The collaborative environment fostered by the CoP offered significant benefits to genetic professionals, facilitating their transition to new practice settings and supporting essential knowledge and skill development crucial for their success in introducing genomics in speciality patient care.

**Supplementary Information:**

The online version contains supplementary material available at 10.1186/s12913-025-12440-2.

## Introduction

Communities of Practice (CoPs) have gained popularity in the health sector as a promising approach to drive knowledge translation, facilitate the implementation and scale-up of evidence-based practices, and advance systems change [1, 2]. A CoP is defined as a group of individuals with a shared concern or passion for what they do [3], who come together to improve their skills and expertise and enhance their practice through regular interactions and collaborative learning [3–5]. The concept of CoPs can be traced back to the work of Wenger [3] based on situated learning theory and adult learning principles. Wenger identified three essential elements of a CoP that need to work well together to create an environment for learning and knowledge generation. These are: (i) the “domain” that creates common ground and common knowledge within the community; (ii) the “community” that facilitates learning through interaction and relationships; and (iii) the “practice” with knowledge and other resources to address problems [3]. A CoP may occur in an informal setting when there is not a deliberate organised approach [3]; alternatively, a formal CoP may be established with intentionality and an organised structured [5, 6]. A CoP is often characterised by peer-to-peer exchanges (rather than hierarchical relationships), an emergent structure, dynamic membership, reciprocal learning, regular interactions, and identity building [3, 5, 7, 8].

To build an effective CoP, the role of an opinion leader or champion and a facilitator has been emphasised [3], as they can provide high-level support and enhance interaction among the group members [8, 9]. Diverse membership from participants with varying levels of expertise and seniority is shown to contribute to experience sharing and team learning [8]. Evidence from successful CoPs has further suggested the use of information technology and different modes of operation, such as virtual meeting platforms, online forums, blogs, social media, listserv, podcasts, and webinars, to promote continuous engagement and facilitate knowledge co-creation both synchronously and asynchronously [9–13].

CoPs have been implemented in healthcare to bring together geographically dispersed professionals to foster collaboration and provide support for career-related growth [10, 14, 15]. Participants acknowledge that CoPs promote self-reflection on their practice [8] and strengthen their professional identity or sense of belonging to the clinical communities [14, 16]. They also note the role of CoPs in providing meaningful networking and mentoring that result in collaboration for funding and employment opportunities [10]. Competence and skill development, for instance, in relation to research skills and evidence-based practices, has also been noted as an important outcome associated with CoP participation [12, 17].

CoPs have been demonstrated as an effective mechanism to facilitate the introduction of new practice and interventions in healthcare setting [18, 19] and there is growing interest in understanding how CoPs contribute to creating change and influencing healthcare outcomes for patients. CoPs can support change by providing an environment that promotes sharing both explicit and tacit knowledge [1, 5], and continuing learning [20], which enables the flow of knowledge at the point of care into larger systems [14]. Through CoPs, the need to support healthcare providers can be identified, leading to the implementation of knowledge-to-action initiatives which stimulate the adoption of change to enhance patient care [9]. Participating in CoPs helps healthcare providers understand the organisational context, which contributes to their capability in solving implementation-related problems and influencing management, administration, and other staff at their workplace to improve care [8]. The cost reduction of clinical practice associated with engaging in a CoP has also been identified as an important driver to healthcare professionals’ participation [21]. Further, when operating at the point of care and service delivery, CoPs have the potential to detect risks early on and recommending mitigation measures prior to any negative consequences or adverse outcomes occurring [14].

Despite the burgeoning literature on CoPs in the health sector, little is known about how CoPs can support healthcare professionals transitioning to new environments with a high degree of uncertainty and limited peer support. In addition, scant attention has been paid to understanding how CoPs stimulate practice changes [14, 15] and there are a lack of studies with rigorous methodologies and theoretical frameworks to guide establishment and operation of CoPs [13, 15]. To address these gaps, our study investigates a CoP for genetic health professionals working in new roles in which they were employed to support the delivery of genomic testing and counselling outside of clinical genetics services. The establishment of the CoP was evidence-based and we deployed structured documentation of the CoP across the cycle of its formation and implementation. The paper explores how participating in this CoP supported its members in their new roles and practice.

## Methods

### Theoretical frameworks

The formation of our CoP was informed by social learning theory which considers human learning outside of the individual and posits that new behaviours, skills, and attitudes are learned through social interaction (including observation and modelling) [22]. This particular study which focuses on the impacts of the CoP on participating genetic counsellors (GCs) was guided by the integrated Promoting Action on Research Implementation in Health Services (i-PARIHS) [23], a refined version of the PARIHS framework [24]. The i-PARIHS framework recognises the complexity characterising the process of implementing evidence-based practices and states that successful implementation requires strong alignment between four elements: innovation, recipients, the context in which the evidence is implemented, and appropriate facilitation of the implementation process. Facilitation—referring to how innovations are supported in clinical practice—is both a specific role and a process of enabling the implementation, which is a unique feature and the active ingredient in i-PARIHS. Three skill areas emphasised in the facilitator attributes include: project management and improvement; team and process; and influencing and negotiating [23]. In our study, we focus on this construct to understand how the facilitation of a CoP external to the implementation setting [25] can support health professionals performing their roles in new working environments (*the new role was conceptualised as a new practice or an innovation*). We also acknowledge the second dimension of facilitation characterising the new roles of GCs participating in this CoP: those professionals were facilitating the use of genomics through dedicated positions in non-genetic health disciplines.

### The Australian context and the genetic counselling community of practice

In Australia, Genetic Counsellors are allied health professionals who have completed a two-year Master’s degree in genetic counselling as the entry-to-practice requirement. Associate Genetic Counsellor are Masters qualified, early career GCs working towards certification to become a Certified Genetic Counsellor [26]. GCs in Australia are mainly employed by clinical genetics services funded by the relevant Australian state or territory health system. However, new positions have emerged for GCs to work in non-genetic specialities, such as in new models of care in oncology and reproductive genetics [27], and private clinics [28]. While working outside of clinical genetics services presents numerous opportunities, genetic health professionals can face a range of challenges in new work environments [29].

The CoP under study was established in May 2023 with three main purposes: providing peer support, experience sharing, and learning for GCs working in ‘mainstream roles’ – positions outside of clinical genetics services. The GCs were employed by different hospitals with funding provided by a Change program to work in multiple clinical change projects. These projects set up innovative models of care in various medical specialities, including neurology, neuropsychiatry, nephrology, haematology, transplant, cardiology, and paediatrics [30]. 

**Fig. 1:**
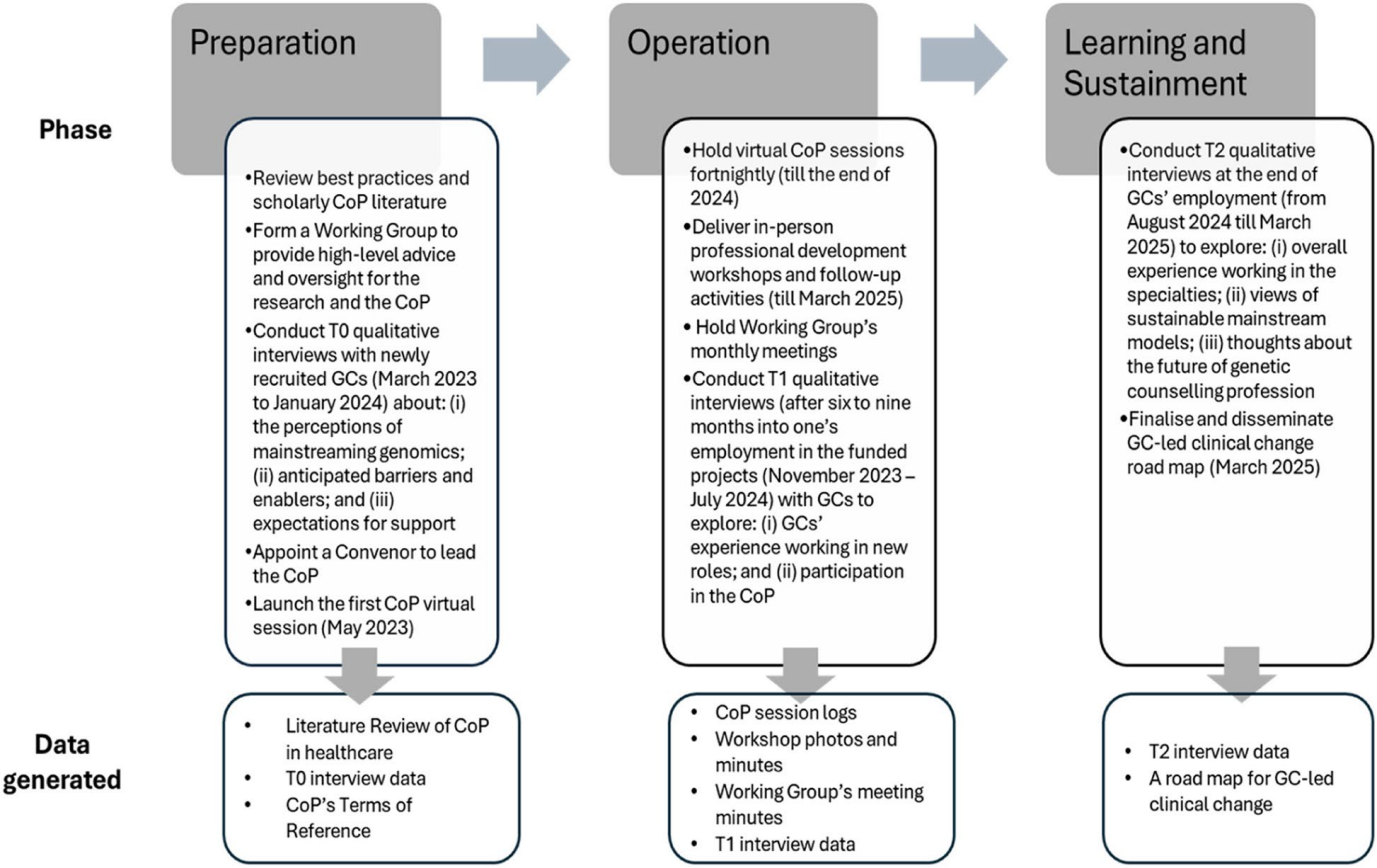
The main phases of establishing and operating the CoP

The Change program includes an overarching research study to understand what is required to support and sustain the use of genomics in an Australian, publicly funded health system. A sub-study exploring the changing roles of GCs in response to the growing demands for genomic healthcare was included. Initial interviews (T0 interviews) were conducted with all GCs in the clinical change projects, which informed the establishment of this CoP [30]. In return, discussion within the CoP served to guide T1 and T2 interviews within the sub-study (Fig. 1 illustrates an overview of activities across different phases of the CoP development).

With a focus on the new ‘mainstream roles,’ the CoP was separate from supervision—a professional activity required for all GCs in Australia throughout the duration of their careers [31]. Focusing on the counselling practice, supervision serves educational and supportive functions and is sometimes referred to as reflective practice supervision or professional supervision. Supervision is essential for genetic counselling practice, training, and certification to ensure the safety of GCs and their clients and mandated by the Human Genetics Society of Australasia (HGSA) in the Competency Standards for Genetic Counsellors and Code of Ethics for Genetic Counsellors. The CoP offered through the Change program should be seen as a complementary activity rather than a substitute of ongoing supervision arrangements for its members. While participating in the CoP was voluntary, every GC was encouraged to join at the start of their new role and consider this as part of their funded position in the change project. A senior GC and educator was appointed to lead the CoP (the *Convenor*) and become a member of the research team (the *Working Group*), but was not employed in any of projects funded by the Change program. The CoP’s Terms of Reference were co-developed by its members, which provided the principles for an evolving structure and dynamic focus that aligned with the needs of the group and the requirements of their roles in the medical specialities (See Supplementary 1). The group set out to meet online for an hour fortnightly and held professional development workshops in person. Each participant attended an average of 11 sessions. The CoP sessions focused on four main areas: funded project operation; implementing a new model of care; clinical practice; and skill development (See Supplementary 2).

### Data collection

We collected data from multiple sources at various timepoints across the cycle of the CoP. Qualitative interviews were conducted to explore GCs’ experiences throughout their employment in the medical specialities, which comprises the main data analysed in this paper (the Interview Guide is included in Supplementary 3). Interviews included questions about participants’ expectations for and activities in the CoP, and the perceived impacts of the CoP facilitation informed by i-PARIHS. Each interview lasted from 30 to 50 min and were transcribed verbatim.

In addition, we analysed the Convenor’s logs which were written immediately after each session (a CoP session log),^1^ workshop notes, and minutes of the monthly Working Group meetings. Several questions^2^ recommended for ‘periodic reflections’ in implementation [32] were used to guide the note-taking process to ensure documenting the nuanced discussion, the associated outcomes, and the Working Group’s recurring reflection, learning and actions (see an example of the Research team’s learning and actions provided in Supplementary 2). At the time of writing, 35 virtual one-hour sessions (the last session included in the analysis occurred on 23 September 2024) and two in-person professional development workshops (each run for four hours) had taken place. The two workshops run respectively in November 2023 and July 2024: the first workshop focused on developing an ‘elevator pitch’ to articulate the role of GCs in clinical care while the second workshop used a co-design activity to create a roadmap for genetic counsellor-led clinical change. All notes from the CoP sessions, workshops, and minutes of the Working Group meetings were de-identified and collated.

### Data analysis

Given a lack of definition of the sub-elements under the i-PARIHS Facilitation construct, we used thematic analysis [33] to capture the patterns and process of facilitation beyond describing the activities performed by this CoP. Anonymised transcripts and interview notes were imported into NVIVO 14 software [34] and analysed through six phases (familiarisation through writing interview notes and transcribing; coding with respect to how the facilitation of the CoP supported its participants in doing their new roles; constructing initial themes; reviewing themes against i-PARIHS; defining themes; and writing up). TD, MM, and BM co-coded five transcripts, discussed and resolved coding discrepancies, and agreed on a codebook. Once the codebook was finalised, TD applied it to analyse the remaining interviews, as well as the workshop notes, session logs, and crosschecked with the minutes of the Working Group meetings while remaining open to emerging themes/sub-themes. Throughout this process, the Working Group held regular meetings to review the emerging themes and consider how the facilitation skills proposed in i-PARIHS [23, 35] were reflected in the data.

### Participants

In total, 14 GCs (out of 15 CoP members) participated in our T0 interviews, including twelve women and two men, and 11 of those participants were again interviewed at T1. The recruited sample possessed a diverse range of working experience (Table 1). Among them, 13 participants continued to work at one or more of four clinical genetics services in an Australian metropolitan city while being employed for a 12- or 18- month period in a part-time role in the medical specialities which they were interviewed about. One participant worked exclusively in the change project.

**Table 1:** Participant characteristics

	**Number of participants** (total *n* = 14)
Certification	
Certified	7
Associate	7
Years of experience working as a GC (by the first interview)	
Less than 5 years	6
5 to 9 years	4
10 years and above	4
Primary affiliation	
Public genetics department	13
Private service	1
Mainstream work setting	
Cardiology	1
Haematology	1
Nephrology (adult and paediatric)	6
Neurology/neuro-psychiatry	3
Paediatrics	1
Transplant	2

## Results

From analysing the data, we developed five overarching themes that captured the impacts of the CoP and are presented below. We illustrate the data from the interviews with the Participant code while data from the workshops are reported collectively as a group. Data from other sources are specified as “CoP session” or “Working Group meeting.” Where a name of a speciality area, a person, or an organisation was mentioned in a quote, we replace it with a general term (left in italicised text in selected quotes) to protect the anonymity of participants or the clinic they were employed by.

### Connecting and empowering

The most prominent and recurring theme emerging in many interviews was the meaningful connection with peers working in mainstream roles through the CoP, which offered GCs a great source of emotional support. Participants acknowledged the unique role of the CoP in forging peer connection among these genetic professionals, helping them overcome the feeling of isolation:

[K]nowing that I have that forum and knowing that even though I'm technically on my own for this project, I'm not on my own more broadly because there's a team of people as well. So I think I've gained from having that structure. (#11)

This aspect was perceived as particularly important for those employed as the only genetic professional in a medical speciality clinic:

There are two things: to remain feeling connected to people doing similar work because I do a little bit on my own, out in the *speciality* space. (….) [I]n the beginning, that was a little bit harder. (#07)

Through the CoP, participants acknowledged the distinct nature of their new roles and started to identify themselves with peers in this group, thereby developing a sense of unity with other GCs based on the shared traits of working in medical specialities:

[T]he other genetic counsellors... they don’t specifically understand what you are talking about. I have regular group [genetic counselling] supervision but everyone in my group works clinically in genetics, not in mainstreaming. So the challenges that they’re having and we’re having [are] a little bit different. It’s great to have a group with everyone who’s involved in mainstreaming. (#03)

The meaningful relationships developed through the CoP enabled participants to draw strength and support from their peers, which was critical for them to persevere through difficult workplace situations. In addition, they perceived the CoP as an effective channel for mutual learning and experience sharing.

### Facilitating co-learning and sharing knowledge of innovations

The structured learning environment characterising this CoP was well-received among its members who believed they benefited from ongoing knowledge acquisition about good practices and innovations. Most notably, staying abreast of good practices implemented by other GCs was among the major benefits participants perceived to have gained from this environment:

I felt I could learn a lot from hearing their experiences and ideas that I could take to *Hospital 1*... [E]veryone just brings their own opinion. I feel where everyone can have different opinions on how their cases [were] or they approached cases or things when we were setting up a new project. (#08)

The CoP also encouraged GCs’ openness to new ideas and willingness to implement innovations in their practice. A participant described how they put new learnings into practical use within their own project thanks to the knowledge gained from the CoP:

I asked the project team to put together a Teams [Microsoft Teams] channel, all the *speciality* genetic counsellors to be included in so that we could share resources and chat with each other … so that was something that I thought I discussed at the CoP would be really helpful.” (#06)

For others, knowledge co-creation was achieved by sharing and discussing interesting cases or useful resources among this group. For instance, participants recognised the relevance of the explicit knowledge sourced from the scholarly literature disseminated within the CoP:

[T]his particular one [a paper presented] was what sorts of things should managers be aware of to support genetic counsellors working in these types of position… these are some of the things that I think will be helpful for me. (#07)

The members did not merely perceive themselves as passive learner in this environment, they also recognised their ability to contribute knowledge to creating a mutual sharing space:

I was conscious that I had been working in *speciality* genetics for a long time. (…) Some of the genetic counsellors who work at the other sites perhaps didn't have as much *speciality* genetics experience or hadn't been working as long as a genetic counsellor and so just being able to contribute some of that experience as well. (#06)

In summary, participants valued the CoP as a unique environment that fostered ongoing learning of good practices and encouraging implementation of innovations in the context of GCs’ mainstream roles. Further, the CoP provided an effective space for identifying and solving problems collectively, as we present in the next theme.

### Offering a collective problem-solving space

The CoP enabled practice improvement efforts through offering a forum for problem identification and solving. In the CoP sessions, participating GCs often shared the challenges encountered at different stages of their new roles. As a group, they worked collectively to generate a solution. For instance, in the first few months of the CoP when most of its members started to be introduced to the medical speciality team, the Convenor noted in their discussion logs:

We brainstormed and discussed about ways to increase visibility in clinic environments where there is nowhere to sit so the GC feels like they are ‘loitering’ and ‘wasting time.’ We talked about going to the clinic for 30 minutes each time – being present, checking in, handing the physicians a card with their number, and attending the weekly multidisciplinary team meetings that all physicians attend. (CoP session - 4 July 2023)

Further, in our interviews participants often described how they harnessed the wealth of knowledge held by this group by applying the lessons learnt across different projects and identified areas to avoid duplicating efforts:

[T]he early days we were talking about setting up processes. I think it stopped this double handling things…. I talked about things that I'd done (at mainstream site) and set up and so we didn't have to do them twice. (#08)

When an implementation issue arose and could not be resolved within the CoP, the participants recognised the role of the Convenor in escalating clinical and practical issues to the organisational leadership for follow-up and further action:

That [CoP] was a really nice resource to be able to access, and it felt really safe place … and the support from the *Convenor* with liaising with the *Change program* for the issues that we were having, so [the Convenor] not just removing herself and saying: “I'm not going to get involved,” [but] actively helping us workshop some of these issues. (#06)

As evidenced in the aforementioned quote, when emergent issues were escalated to the management team through the Working Group, they prompted the leadership of the Change program to respond in a timely way to the challenges that may hinder the GCs’ progress. In this way, the CoP enabled leadership decision-making and eliminated the risk of inaction.

### Improving interpersonal skills to strengthen inter-professional collaboration

As GCs’ new roles in medical specialities substantially involved collaborating with non-genetic professionals, this CoP paid greater attention to the aspect of forging inter-professional collaboration. This focus was consistent with earlier interviews with this group of GC participants who noted the tension (potentially) arising in this collaboration due to the differences in the clinical approaches between genetic and non-genetic disciplines (Authors, 2024). Hesitance among some non-genetic colleagues about integrating genomics in their clinical practice was also anticipated at the early stage of implementation and frequently appeared in the CoP sessions:

The more recent GC came today, she said she is “not making any friends [experiencing a lack of engagement] among the specialists.” There was a comment about fluctuating confidence in what the GC can expect specialists to upskill in and what not. (CoP session - 5 May 2024)

In view of this, throughout our interviews, participants often described specific examples of how they could learn collaborative skills and lessons to develop better engaging approaches to increase their colleagues’ awareness of genetic counselling support and influence the practice of using genomics in the speciality clinics:

[T]hey [other CoP members] have been sitting in clinics, but people [physicians] weren't coming to them directly, so they set up their own registrar training sessions. And [I] thought it was great. So I picked up on [it] myself at *Hospital 1*. Other GCs [in the CoP] are more clinically experienced than I am... I've learned a lot from hearing how they've approached it and their ideas. (#08)

Participants also believed that the CoP discussions enabled them to better manage their expectations and become more mindful of practice boundaries when working in multidisciplinary teams. For example, in one case, a GC had previously thought that they should not overstep the boundaries because the patients were “their [physicians’] patients.” Later, they shifted to accept the need to influence changing the practice of non-genetic colleagues. Participant #03 noted:

I find *the Convenor* really helpful, just advice and having a forum to raise, for example, when I felt like I should play more of a passive role, then it could be one that I could be active. (#03)

In addition, some participants reported developing the confidence critical for them to advocate for themselves while working with more senior colleagues who might hold a different clinical approach. This was most resounding among early-career members, as a GC who was a recent graduate said in one interview:

[T]hat session and that conversation with the team helped me feel a bit more confident to come back to *Physician* and explain what my concerns were and how I couldn't mitigate those. So I think [it was] helping me feel confident that I can speak up and be an advocate when I feel like there's something to bring up. (#11)

### Shifting the perspectives of an evolving role through critical reflection

Through diverse activities and interaction with peers and the Convenor, participants regarded the CoP as an effective forum for critical reflection, which helped them view the mainstream role from different angles and offered future-oriented thinking for the genetic counselling profession. Many participants reported that being more reflective of their current work made them better understand the implementation context, as Participant #05 said:

[I]t is an extra chance to touch base and hear how things are going at other sites. And is it similar to what we're doing? Is it different to what we're doing? And is that a problem? (#05)

Hearing diverse experiences and views made participating GCs feel validated in their own perspectives and approaches. For instance, one GC recalled a CoP session when they discussed a complex situation of disagreement with the physicians about offering genomic testing to a young patient:

I brought that [case] a couple of times and often asked for help with that and it was helpful for people to reinforce that my concerns were valid about making sure this young person is making an informed decision that is not going to be too harmful to [them]. (#11)

Finding emotional validation was especially beneficial when a GC started a new role with a high degree of uncertainty about how their role would be perceived and integrate into the medical speciality. For them, knowing about their peers’ experiences helped normalise challenges and frustrations they could encounter in their role. This helped them overcome the discomfort and persist throughout challenging times:

There's been times where I've felt a bit uncomfortable … where perhaps maybe a clinician, maybe I don't feel as if they're taking on board my genetics contribution as much as someone else, having that validated by all the experiences that are happening with everyone else has really let me be able to just kind of sit in that moment anyway and know that I'll move through that and then just like other clinicians, that clinician, possibly in the future, might shift a bit as well. (#01)

In addition, regular reflective practice facilitated through the CoP helped GCs contemplate their sphere of control when working outside of clinical genetics. By doing so, they were able to identify actionable areas:

It's about further engagement, and how we focus on what we can control versus not. We can't control the number of patients being referred. But we can control how involved we are in team meetings and the follow-ups that we make. (#02)

The CoP discussion and activities prompted its members to formulate thoughts about the future of the genetic counselling profession, as shared by a participant after a professional development workshop: “We did exactly what I hoped for, which is open collaboration to envision the future of mainstreaming. I hoped to feel invigorated and inspired by the conversations, which I do.”

With increased awareness of the broader implications for their future work, some reported taking action to advocate for longer-term positions for GCs in the medical specialities to sustain the integration of genomics in routine care, as one participant expressed:

[T]he project ends at a certain point, but hopefully it's not going to be the end of all of this. And that I can see and I've said to the *physician* team here as well, maybe it means that they need to think about a role for genetics and a genetic counsellor ongoing in their department and the different models that might come out of this. (#09)

In summary, with regular reflective activities and conversations happening within this platform, the CoP contributed to redefining mainstream practice, helping participating GCs deepen the meaning of their new roles and better prepare for the realities of working in the medical specialities.

## Discussion

Our study describes the formation and impacts of a CoP established for GCs employed to deliver innovative models of providing genomic testing and counselling in medical specialities. Findings of the present study have revealed multiple benefits of the CoP, highlighting the rich learning experience, new knowledge of good practices, skill development, and meaningful peer support through regular group discussions and in-person professional development workshops.

Our findings expand the understanding of the Facilitation construct conceptualised in the i-PARIHS framework [23] by showing the value of the CoP in providing emotional support to health professionals starting their new roles. This aspect is often under-emphasised in implementation studies unpacking the facilitation role guided by i-PARIHS [25, 35] and is only noted in a small handful of studies in the CoP literature [10, 36] where more attention is placed on the benefits regarding professional growth and practical support for its members. In our study, the participants highly valued the emotional support resulting from the peer connections forged within the CoP, since it was conducive to overcoming the feeling of working in silos in medical specialities and with professionals from other disciplines. Participating GCs also found a sense of unity through regular interactions with their peers within the CoP, which carried them through challenging times and frustrating situations. The emotional benefit should be sufficiently acknowledged when considering the role of facilitation to help reduce stress and feeling of professional isolation [36], thereby enhancing job satisfaction and success of healthcare professionals working outside of the familiar environments to foster change into clinical settings.

A unique focus of our study is to understand the impact of the CoP—as external facilitator to the implementation setting—on GCs’ new roles to introduce change into genomic care pathways. Our study supports the description of the facilitation attributes in i-PARIHS in relation to project improvement, and process and team skills [23]. Specifically, our findings suggest that the information exchange facilitated through this CoP enabled its members to learn from observing and discussing others’ experience in similar situations and facilitated the learning and adoption of good practices to solve implementation issues uniquely encountered in mainstream roles. In doing so, we demonstrated how the principles of social learning could explain the diffusion of innovation [37] and support improvement efforts. In addition, the CoP provided a space for building inter-personal skills to overcome relational challenges and improve communication with non-genetic colleagues about genomics, thereby solving situations of disagreement or tension and overall forging interprofessional collaboration in multidisciplinary teams. The impact on interdisciplinary collaboration is often observed in multiprofessional CoPs [6, 38] and was also noted in this study, despite the context being a uni-professional CoP. This is likely due to the intention underpinning the establishment of the CoP to support mainstream GCs whose roles required regular interaction with non-genetic professionals to support the integration of genomics into routine care. Therefore, for organisations aiming to leverage this structure to facilitate the implementation of new practices, we recommend CoPs that are intentionally formed [6, 14], offer protected time for attendance, and are formally endorsed by the leadership.

A prominent finding of our study is the emphasis on reflection as both a practice and an impact of the CoP. We found an interactive effect between critical reflection and giving meaning, the two sub-elements under skills and attributes within the Facilitation construct suggesting a ‘high’ chance for successful implementation in the original PARIHS framework [24]. Specifically, being more reflective through the CoP sessions and activities helped GCs reshape the meaning of their roles in mainstream settings. The individual and collective reflection encouraged through this CoP environment also resonates with the reflective practice that is common in the genetic counselling profession [39] and considered as a core factor of effective learning [40, 41] and clinical leadership [42]. In the present study, participants saw the interconnection between the work they were doing across projects and sites, thereby enabling intersubjectivity and enhancing the effectiveness of knowledge co-production [43]. This reinforces the ‘think together’ process [44], as it allows participants to draw on both objective knowledge and subjective views and experience to transform their learnings to adapt to different social and practical contexts rather than passively receiving knowledge from others.

Existing research has demonstrated identity-building among the central characteristics of many CoPs [5, 45] due to strong connectivity of their members, which serves to differentiate this structure from other forms of knowledge communities [46]. In our study, it is not yet clear whether a new identity of ‘mainstream GCs’ has been adopted among the members of this CoP because the scope of their roles in the medical specialities were still evolving at the time of our interviews. The diverse prior clinical experience that the GCs possessed may also affect the dynamics of identity formation. This CoP included early career GCs who were still in the transition from training to practice and may experience professional identity evolution [47, 48]. Meanwhile, more experienced members might find themselves negotiating an already-existing identity [49], rather than creating a new one, as a result of working in new roles and environments. For those participants, their professional identity may be tied to one’s educational background and shared values, rather than the physical location where GCs practised. We, however, note that the GCs interviewed in this study started to identify themselves with the peers in the CoP group: they articulated the commonalities across different health services and funded projects that were not experienced by those working solely in clinical genetics settings, which may result in a disconnect between their individual identity and the collective identity of the genetic counselling profession [50]. Those shared characteristics, including the similar nature of mainstream work and its relatable frustration and challenges may constitute the new ‘identity markers’ [51] when a greater number of GCs transition to working in medical specialities in the future.

### Strengths and limitations

The strength of our study is the focus on an on-going CoP and the deployment of structured data collection since inception, covering both the reflection of the Convenor and data from the recipients of the facilitation through interviews and other activities. This provides an opportunity to address some of the methodological challenges noted by previous studies applying i-PARIHS [25], as we could triangulate the data from multiple sources, follow up on topics of interest, and document the continuity and dynamic development of CoP discussions, and the outcomes of resulting actions. In addition, analysing data from different sources over different timepoints enables us to capture the nuances of the knowledge transfer process in the practical context of the funded projects across different health services, which is often challenging when studying learning organisations [46]. It also helps address the recollection bias when participants in the qualitative interviews were unable to recall the accounts of their participation since the early days of the CoP.

To address the limitations in terms of time and finance that have been found by others to constrain CoP participation [14, 45], the current CoP was designed for participants who had paid time to participate, as their positions and that of the Convenor were funded. Because all those roles were time-bound, this CoP might not continue when the current change projects end. However, given the positive outcomes perceived by the participants in this study, we are hopeful about the emergence of similar platforms. One example informed by the principles and structure of the CoP described here had already been adopted by Participant #06 in their own project. It is also worth noting that this CoP was designed for GCs and facilitated by an experienced educator from the same profession for whom reflection is an integral component of their professional practice [39, 50]. Therefore, the finding related to critical reflection might take longer to achieve among other professional groups who are not as well acquainted with a reflective approach. We recommend future research to investigate the long-term impacts of the CoPs on professional practice and the adoption of a new professional identity. Additionally, capturing how health organisations’ leaders perceive and interact with these learning structures could provide valuable insights for broadening the CoPs’ impacts on systems change.

## Conclusion

This paper has explored the formation and impacts of a CoP for mainstream GCs. The CoP has proven to be a valuable platform for its members, significantly enhancing their connectivity, offering emotional support, and providing effective solutions to challenging situations or tension arising in their new roles in medical specialities. The emphasis on reflection, both as a practice and as an impact, has been a critical characteristic of this CoP, which enables its members to find emotional and intellectual validation and apply their learnings into practical contexts. Particularly for health professionals transitioning to new ways of working, our findings emphasise the pivotal role of supporting the implementation of CoPs, as the collaborative environment and shared learning experiences promoted within this platform can ease their transition and advance professional growth.

## Supplementary Information

Supplementary Material 1.

Supplementary Material 2.

Supplementary Material 3.

## Data Availability

The datasets generated during the current overarching study are not publicly available as data collection is still ongoing, but the data analysed in this paper are available from the corresponding author on reasonable request.

## Abbreviations

CoP: Community of Practice
GC: Genetic Counsellor
PARIHS: Promoting Action on Research Implementation in Health Services

## Footnotes

1 The detailed content of all CoP sessions was kept confidential from the rest of the research team as outlined in the Terms of Reference (see Supplementary 1).

2 These questions include: 1) Have there been any surprises lately, or unexpected events? 2) Have barriers/concerns arisen recently? 3) What solutions have been trialled? 4) What are the main suggestions going forward? 5) What are the next steps going forward? and 6) What are the key learnings?

## References

[1] Ranmuthugala G, Plumb JJ, Cunningham FC, Georgiou A, Westbrook JI, Braithwaite J. How and why are communities of practice established in the healthcare sector? A systematic review of the literature. BMC Health Serv Res. 2011;11(1):273. 21999305 10.1186/1472-6963-11-273 PMC3219728

[2] Livergant RJ, Ludlow NC, McBrien KA. Needs assessment for the creation of a community of practice in a community health navigator cohort. BMC Health Serv Res. 2021;21:1–12. 34225704 10.1186/s12913-021-06507-z PMC8256652

[3] Wenger E. Communities of practice: A brief introduction. 2011.

[4] Coghlan D, Brydon-Miller M. The SAGE encyclopedia of action research: Sage; 2014.

[5] Li LC, Grimshaw JM, Nielsen C, Judd M, Coyte PC, Graham ID. Use of communities of practice in business and health care sectors: a systematic review. Implement Sci. 2009;4(1):1–9. 19445723 10.1186/1748-5908-4-27 PMC2694761

[6] Brooks SP, Ekpe Adewuyi E, Wasylak T, Thomson D, Davison SN, Storey K. How to use communities of practice to support change in learning health systems: A landscape of roles and guidance for management. Learning Health Systems. 2024:e10412. 10.1002/lrh2.10412 PMC11257050 39036528

[7] Jeon S, Kim YG, Koh J. An integrative model for knowledge sharing in communities-of-practice. J Knowl Manag. 2011;15(2):251–69.

[8] Sahay A, Mittman BS, Gholami P, Lin S, Heidenreich PA. How successful was the use of a community of practice for the implementation of evidence-based practices for heart failure within the United States Department of Veterans Affairs: Insights from a formative evaluation. Health Research Policy and Systems. 2022;20(1):79. 35804413 10.1186/s12961-022-00880-9 PMC9264639

[9] Kothari A, Boyko JA, Conklin J, Stolee P, Sibbald SL. Communities of practice for supporting health systems change: a missed opportunity. Health Research Policy and Systems. 2015;13(1):1–9. 26208500 10.1186/s12961-015-0023-x PMC4515005

[10] Fix G, Seaman A, Nichols L, Ono S, Rattray N, Solimeo S, et al. Building a Community of Anthropological Practice: The Case of Anthropologists Working within the United States’ Largest Health Care System. Hum Organ. 2023;82(2):169–81. 10.17730/1938-3525-82.2.169 PMC13450104 42569530

[11] Mier-Alpaño JD, Cruz JRB, Fajardo MS, Barcena JF, Ekblad E, Hazell F, et al. Facilitating learning exchange and building a community of practice to accelerate social innovation in health. BMJ Innovations. 2022:bmjinnov-2021.

[12] Keir A, Bamat N, Hennebry B, King B, Patel R, Wright C, et al. Building a community of practice through social media using the hashtag# neoEBM. PLoS ONE. 2021;16(5): e0252472. 34048469 10.1371/journal.pone.0252472 PMC8162580

[13] Shaw L, Jazayeri D, Kiegaldie D, Morris ME. Implementation of virtual communities of practice in healthcare to improve capability and capacity: a 10-year scoping review. Int J Environ Res Public Health. 2022;19(13):7994. 35805649 10.3390/ijerph19137994 PMC9265616

[14] Auer AM, Hanson P, Brady-Fryer B, Alati-It J, Johnson AL. Communities of practice in Alberta Health Services: advancing a learning organisation. Health Research Policy and Systems. 2020;18(1):1–12. 32746853 10.1186/s12961-020-00603-y PMC7397570

[15] Noar AP, Jeffery HE, Subbiah Ponniah H, Jaffer U. The aims and effectiveness of communities of practice in healthcare: A systematic review. PLoS ONE. 2023;18(10): e0292343. 37815986 10.1371/journal.pone.0292343 PMC10564133

[16] Jaye C, Egan T, Smith-Han K. Communities of clinical practice and normalising technologies of self: learning to fit in on the surgical ward. Anthropol Med. 2010;17(1):59–73. 20419517 10.1080/13648470903569388

[17] Becerril-Montekio V, Alcalde-Rabanal J, Darney BG, Orozco-Nuñez E. Using systematized tacit knowledge to prioritize implementation challenges in existing maternal health programs: implications for the post MDG era. Health Policy Plan. 2016;31(8):1031–8. 27060787 10.1093/heapol/czw033 PMC5013782

[18] Poissant L, Ahmed S, Riopelle RJ, Rochette A, Lefebvre H, Radcliffe-Branch D. Synergizing expectation and execution for stroke communities of practice innovations. Implement Sci. 2010;5(1):1–8. 20529305 10.1186/1748-5908-5-44 PMC2890694

[19] Barbour L, Armstrong R, Condron P, Palermo C. Communities of practice to improve public health outcomes: a systematic review. J Knowl Manag. 2018;22(2):326–43.

[20] Conklin J, Stolee P. A model for evaluating knowledge exchange in a network context. Canadian Journal of Nursing Research Archive. 2008:116–25. 18714901

[21] Lacasta Tintorer D, Flayeh Beneyto S, Manresa JM, Torán-Monserrat P, Jiménez-Zarco A, Torrent-Sellens J, et al. Understanding the discriminant factors that influence the adoption and use of clinical communities of practice: the ECOPIH case. BMC Health Serv Res. 2015;15(1):1–10. 26358037 10.1186/s12913-015-1036-4 PMC4566431

[22] Mukhalalati BA, Taylor A. Adult learning theories in context: a quick guide for healthcare professional educators. J Med Educ Curric Dev. 2019;6:2382120519840332. 31008257 10.1177/2382120519840332 PMC6458658

[23] Harvey G, Kitson A. PARIHS revisited: from heuristic to integrated framework for the successful implementation of knowledge into practice. Implement Sci. 2015;11:1–13. 10.1186/s13012-016-0398-2 PMC4807546 27013464

[24] Rycroft-Malone J. The PARIHS framework—a framework for guiding the implementation of evidence-based practice. J Nurs Care Qual. 2004;19(4):297–304. 15535533 10.1097/00001786-200410000-00002

[25] Connolly SL, Sullivan JL, Ritchie MJ, Kim B, Miller CJ, Bauer MS. External facilitators’ perceptions of internal facilitation skills during implementation of collaborative care for mental health teams: a qualitative analysis informed by the i-PARIHS framework. BMC Health Serv Res. 2020;20:1–10. 10.1186/s12913-020-5011-3 PMC7057643 32131824

[26] (HGSA) HGSoA. Genetic Counselling Training and Accreditation 2023 [Available from: https://www.hgsa.org.au/Web/Web/ET/Genetic-Counselling.aspx?hkey=975b23ce-fa8e-485a-a7ee-fc88aab9a60d.

[27] Kanga-Parabia A, Mitchell L, Smyth R, Kapoor T, Duggal J, Pearn A, et al. Genetic counseling workforce diversity, inclusion, and capacity in Australia and New Zealand. Genetics in Medicine Open. 2024:101848. 10.1016/j.gimo.2024.101848 PMC11658314 39712954

[28] Dwarte T, Barlow-Stewart K, O’Shea R, Dinger ME, Terrill B. Role and practice evolution for genetic counseling in the genomic era: The experience of Australian and UK genetics practitioners. J Genet Couns. 2019;28(2):378–87. 30629777 10.1002/jgc4.1053

[29] Quinn E, Mazur K. The experiences of UK-based genetic counsellors working in mainstream settings. Eur J Hum Genet. 2022;30(11):1283–7. 35918538 10.1038/s41431-022-01158-y PMC9343813

[30] Do TT, Martyn M, McClaren B, McEwen A, Gaff C. Becoming agents for genomic change: genetic counsellors’ views of patient care and implementation influences when genomics is mainstreamed. Eur J Hum Genet. 2024:1–9. 10.1038/s41431-024-01686-9 PMC11606944 39210048

[31] Human Genetics Society of Australasia (HGSA). Supervision for Genetic Counsellors 2022 [Available from: https://hgsa.org.au/Web/Web/Consumer-resources/Policies-Position-Statements.aspx.

[32] Finley EP, Huynh AK, Farmer MM, Bean-Mayberry B, Moin T, Oishi SM, et al. Periodic reflections: a method of guided discussions for documenting implementation phenomena. BMC Med Res Methodol. 2018;18:1–15. 30482159 10.1186/s12874-018-0610-y PMC6258449

[33] Braun V, Clarke V. Using thematic analysis in psychology. Qual Res Psychol. 2006;3(2):77–101.

[34] Lumivero. NVivo (Version 14). 2023.

[35] Ritchie MJ, Drummond KL, Smith BN, Sullivan JL, Landes SJ. Development of a qualitative data analysis codebook informed by the i-PARIHS framework. Implementation science communications. 2022;3(1):98. 36104801 10.1186/s43058-022-00344-9 PMC9476709

[36] D’Abaco E, Khano S, Giles-Kaye A, Dhaliwal J, Haslam R, Prakash C, et al. Impact of a collaborative model on community clinician confidence in child and adolescent mental health care, wellbeing, and access to child psychiatry expertise. PLoS ONE. 2024;19(9): e0310377. 39312567 10.1371/journal.pone.0310377 PMC11419376

[37] Rogers E. Diffusion of Innovations. 5th ed. New York: Free Press; 2003.

[38] Padilla BI, Kreider KE. Communities of Practice: An innovative approach to building academic–practice partnerships. The Journal for Nurse Practitioners. 2020;16(4):308–11.

[39] Paneque M, Guimarães L, Bengoa J, Pasalodos S, Cordier C, Esteban I, et al. An European overview of genetic counselling supervision provision. Eur J Med Genet. 2023;66(4): 104710. 36731744 10.1016/j.ejmg.2023.104710

[40] Chang B. Reflection in learning. Online learning. 2019;23(1):95–110.

[41] Larsen DP, London DA, Emke AR. Using reflection to influence practice: student perceptions of daily reflection in clinical education. Perspectives on medical education. 2016;5:285–91. 27638391 10.1007/s40037-016-0293-1 PMC5035279

[42] Edmonstone J. Clinical leadership: the elephant in the room. Int J Health Plann Manage. 2009;24(4):290–305. 18770874 10.1002/hpm.959

[43] Nguyen-Trung K, Saeri AK, Kaufman S. Incorporating pragmatism in a behaviour change-led climate adaptation project: a collaborative reflection. Qualitative Research Journal. 2024.

[44] Pyrko I, Dörfler V, Eden C. Thinking together: What makes Communities of Practice work? Human relations. 2017;70(4):389–409. 28232754 10.1177/0018726716661040 PMC5305036

[45] Alary Gauvreau C, Le Dorze G, Kairy D, Croteau C. Evaluation of a community of practice for speech-language pathologists in aphasia rehabilitation: a logic analysis. BMC Health Serv Res. 2019;19:1–14. 31358002 10.1186/s12913-019-4338-0 PMC6664764

[46] Hoadley C. What is a community of practice and how can we support it? Theoretical foundations of learning environments: Routledge; 2012. p. 286–99.

[47] Cornett M, Palermo C, Ash S. Professional identity research in the health professions—a scoping review. Adv Health Sci Educ. 2023;28(2):589–642. 10.1007/s10459-022-10171-1 PMC10169899 36350489

[48] Rasmussen P, Henderson A, Andrew N, Conroy T. Factors influencing registered nurses’ perceptions of their professional identity: an integrative literature review. The Journal of Continuing Education in Nursing. 2018;49(5):225–32. 29701865 10.3928/00220124-20180417-08

[49] Nyström S. The dynamics of professional identity formation: Graduates’ transitions from higher education to working life. Vocat Learn. 2009;2:1–18.

[50] Stenberg K, Mills R, Kalia I, Schwartz L. Genetic counselors' professional identity in North America: A scoping review. Journal of Genetic Counseling. 2024. 10.1002/jgc4.1931 PMC11735250 38860487

[51] Liberati EG, Gorli M, Scaratti G. Invisible walls within multidisciplinary teams: disciplinary boundaries and their effects on integrated care. Soc Sci Med. 2016;150:31–9. 26730879 10.1016/j.socscimed.2015.12.002

