# Supplementary material for: How can a community of practice support healthcare professionals navigating new roles? a case study of genetic counsellors employed to work in medical specialities
Source: BMC Health Serv Res. 2025 Feb 25;25:314. doi: 10.1186/s12913-025-12440-2 (PMC11863419; doi:10.1186/s12913-025-12440-2)
Supplement: Supplementary file 1 — Supplementary Material 1. [file 12913_2025_12440_MOESM1_ESM.docx]

Community of Practice

Terms of Reference

**What is a community of practice?**

A community of practice is a forum for professionals to network through regular interaction. Group members share common interests and develop knowledge and skills through participation.

**Purpose**

The purpose of our community of practice is to provide a forum for peer advice and support and improve the ability of genetic counsellors to support the adoption of genomics in diverse specialty areas.

**Scope**

The community of practice groups for clinical change project genetic counsellors facilitate a place for knowledge exchange, support, problem-solving, and sharing and discussion of celebrations and challenges.

**Guiding principles**

- Open, collaborative meeting place for genetic counsellors participating in clinical change projects.
  - Members are encouraged to attend regularly to facilitate the development of a collegial, supportive community
- Respectful, non-judgemental space for sharing the opportunities and challenges in embedding mainstreaming across a variety of specialty areas
- Members share responsibility for active participation in the community by bringing items for discussion and contributing to collective problem-solving

**Objectives**

The objectives of the Community of Practice are to:

- Offer support to genetic counsellors participating in clinical change projects.
- Provide a space to celebrate successes, explore challenges, identify opportunities and share problem solving for genetic counsellors participating in clinical change projects.
- Foster the ability of the genetic counsellors to support adoption of genomics in specialty areas.

**Meeting details:**

We will meet fortnightly for an hour, via Zoom.

**Members and facilitator**

The community of practice will be facilitated by the Facilitator (name blinded for peer review*).*

The members of the group are genetic counsellors participating in clinical change projects funded by Melbourne Genomics.

The groups are open, with members attending as needed and as time allows.

We expect that prioritising attendance where possible will help to facilitate community building and maintenance, and provide a regular time for discussion with other genetic counsellors.

**The members of the community of practice can expect that the facilitator will:**

- Facilitate provision of a non-judgemental space for reflection and discussion
- Listen
- Behave respectfully
- Model reflective behaviour
- Maintain confidentiality as agreed in the terms of reference
- Keep a brief written summary of each meeting

**The members of the community of practice will:**

- Come to the meetings prepared to share the responsibility for each meeting
- Listen and respond respectfully
- Refrain from judgement
- Maintain confidentiality as agreed in the terms of reference
- Attend meetings in a quiet, private space where you will not be overheard

**Confidentiality:**

All material presented and discussed in the community of practice meetings will remain confidential to the community of practice, except:

a) The facilitator may take any material to her own supervision.

b) Should the facilitator be concerned about issues of safety for either the client, a group member or the organisation, she may break the confidentiality. In this situation she will inform the group member and consult her own supervisor prior to breaking the confidence.

c) The facilitator is a member of the research Working Group. She provides a brief summary of overarching themes from each meeting to the research team. The summary does not include names or specialty areas.

##### **Conditions**

- An agenda will be set at the beginning of each session, and may include a review of any work/tasks/concerns undertaken since the last session.
- The group members will be primarily responsible for bringing the material for each session, but the facilitator may from time to time bring issues or topics to attention.
- As the facilitator is external to the group members organisations, the facilitator will not hold clinical responsibility for the group members’ practice.

**Conflict resolution**

Should conflict or disagreement arise in the community of practice the following steps will take place;

1. The two parties will in the first instance endeavour to discuss and resolve the issue between themselves.
2. If the above fails an outside mediator, who is acceptable to both parties, will be called in to help resolve the situation.

**Review**

- The terms of reference will be reviewed and verbally confirmed within three months of being initially discussed
- Thereafter the community of practice will be reviewed annually, or at any time as requested by either party
