# Supplementary material for: How can a community of practice support healthcare professionals navigating new roles? a case study of genetic counsellors employed to work in medical specialities
Source: BMC Health Serv Res. 2025 Feb 25;25:314. doi: 10.1186/s12913-025-12440-2 (PMC11863419; doi:10.1186/s12913-025-12440-2)
Supplement: Supplementary file 2 — Supplementary Material 2. [file 12913_2025_12440_MOESM2_ESM.docx]

**Supplementary 1.** **Documenting discussion and actions**

| **Domain** | **Examples**  **[Extracts: Source of Data, Date]** |
| --- | --- |
| Operation of funded projects | The other issue also raised during this session was related to the ‘project panic’ due to the shift to expediting patient recruitment to the research study. (CoP session - 6 May 2024) |
| Implementation of new models of care | We discussed: Clinicians are sceptical that test has utility (but will order because patient requested it). So there is a role of genetic counsellors in helping clinicians see that ‘utility’ includes family utility, not only whether or not the test will result in a change in clinical management (CoP session - 16 October 2023) |
| Clinical practice | What a ‘complex’ patient is and how this would be defined if the GCs were to see the ‘complex’ patients - who can be considered as ‘complex,’ whether based on diagnosis, the test results or the psychosocial needs of the case. (CoP session - 26 June 2023) |
| Skill development | After someone presented a published paper on GCs working in mainstream roles in the U.K, we also talked about skills and attributes for GCs in these roles, including: strong skills in building relationships; good initiative; access to alternative support and supervision; access to a specialist champion; focus on autonomy; and the ability and confidence to justify role and presence. (CoP session - 14 December 2023) |
| Research team’s learning and actions | We pointed to the need of considering: What genetic counselling will look like in the future? How do GCs conduct themselves and take on ‘new way of thinking’? It’s important to make GCs feel comfortable with the differences they experience in their new roles that they are not familiar with in their clinical roles. (Working Group meeting - 3 July 2024) |
