# Supplementary material for: How can a community of practice support healthcare professionals navigating new roles? a case study of genetic counsellors employed to work in medical specialities
Source: BMC Health Serv Res. 2025 Feb 25;25:314. doi: 10.1186/s12913-025-12440-2 (PMC11863419; doi:10.1186/s12913-025-12440-2)
Supplement: Supplementary file 3 — Supplementary Material 3. [file 12913_2025_12440_MOESM3_ESM.docx]

**Supplementary 3. Interview Guide**

**Timepoint 0 Interview**

To begin with, may I ask you a few demographic, background questions?

- What is the highest level of education you have completed?
- At which hospital and department do you mainly work?
- What is your professional role/job title?

1. Can you tell me a bit about your current or most recent position in the genetics service/department? How long have you been working in that role? What are your main tasks?
2. To which service/clinic/clinical specialty will you be appointed/provide support when you are working in this new role? Where will you be physically located? When will you start? How many days per week will you work in your new role? Will you continue to be working in any other roles?

*Prompts:*

- Have you ever worked in a role where you are outside of a clinical genetics service? If yes, what was your experience like?

1. How did you know about this new role? What attracted you to apply for this (new) role?
2. The term mainstreaming is being used a lot at the moment, what’s your understanding of ‘mainstreaming’?

*Prompts:*

- What do you think is the role of genetic counsellors in mainstreaming?
- Speaking of the new role you are in/starting in the Clinical Change Project, would you describe it as mainstreaming role or describe it in another way?

We’re interested in your thoughts at this early stage but recognise you might not know all these details yet. I’ve got a few questions about your new role:

1. What will you do in your new role?
2. What do you expect to achieve in this role? What does success look like in this role?

***Perception of the new GC model***

Thinking about the specialty you are going to work with, what do you know about the current pathways for patients to access genetics?

1. What do you understand are the current challenges with accessing genetics for the patients at the moment?

How will your new role (at/with the clinical specialties) contribute to addressing those challenges?

How will your role contribute to creating genomic change in healthcare in general?

***Anticipated implementation***

1. As compared to your current/previous roles in the genetics service/department what changes do you expect to see when working in your new role?

*Prompts:*

- Is there any changes do you expect to see in the way you interact with the patients?
- Any changes for the patients?
- Any changes for the hospital/clinic?

We’re interested to know more about your interaction with the health professionals when you are going to work with them in your new role. What do you want the health professionals in the specialties to know about your role?

*Prompts:*

- Have you had any prior experience working with non-genetic health professionals? What worked well? what didn’t? How will you work with them in your new role?
- How will your interaction with them impact their use of genomics in dementia/nephrology/transplant (e.g attitudes, confidence, skills, and expertise)?

1. Do you anticipate any challenges or difficulties in your new role?
2. What do you do to get prepared for this role?

In your new role, when you have a complex case, who will you go to seek professional advice/support? Do you have a supervisor? How do you work with them?

How will you maintain the connection with your colleagues in the genetics service/department?

1. What practical or professional support do you need to successfully work in this new role?
2. The Change Program is going to establish a Community of Practice to bring together GCs working in different alternative models of care outside the clinical genetic services. The purpose of the Community of Practice is to provide those genetic counsellors with peer support and learning opportunities. What are your expectations for the Community of Practice?
3. What can the Community of Practices do to support GCs working outside the clinical genetic services professionally and practically?
4. What needs to be done/support is needed to sustain implementing genomics in clinical care?

***Closing***

Is there any other subject or topic you think we need to discuss to better understand your new role?

**Timepoint 1 Interview**

**Introduction**

May I confirm with you: when did you officially start working in the medical specialty? How many days a week do you work in this role? Do you continue working with the genetics department?

1. Where/which sites do you work? What is a typical day like for you when you work in this role?
2. What are the differences in the scope of work have you experienced in this role compared to your role in clinical genetics service/department?

***Experience working in the new role***

1. I’d like to find out about how things are progressing in your new role in the speciality. Can you tell me: What have been the biggest impacts of your role so far?

*Prompts:*

- What changes have you seen for the patient care?
- How about the specialty clinic?
- How about the non-genetic specialists at the clinic (regarding their view on the role of genomics in their work – beyond just attitudes)?
- How about for yourself, your practice, and your views on the value of genomics in specialty care?

1. We’re particularly interested to know more about your interaction with the specialists there. How have you worked with them in your new role?

*Prompts:*

- Do you feel it’s a collaborative and welcoming environment at the specialist clinic?
- What is working well? What isn’t? Can you please provide some examples?

1. You mentioned in our first interview that […] is the challenge when working in the specialty? How is the situation now?
2. Have you encountered any other challenges in this role?

*Prompts:*

- What did you do when you have a complex patient case?
- Have you had any difficult interactions with pts or medical specialists?
- What have you done to manage these? Did you seek advice/support? From whom?
- Did you alter the processes/way of operating? How?
- How do you find about working a dual role between the genetics service and the specialty?

1. How do you find the support you’ve received so far (from the clinician team, site lead, project lead, and the Change program) in your new role? What else do you need to succeed?

***The Community of Practice***

1. Now I’d like to ask you about your participation in the Community of Practice (CoP). When did you first join the CoP? How often do you attend?
   - 1. If regular, what attracts you to the CoP?
     2. If not regular, what are the barriers to your participation?
2. What have you gained from participating in the CoP?
3. What have you contributed to the CoP?
4. Have you used any skills, knowledge, or experience you learn from the CoP in your new specialty role, or in clinical genetics role? In what ways? Do you have any examples you could share with me?
5. What is working well? What isn’t? What would you like to change about the CoP?

***Sustainability?***

1. Given what you’ve experienced and learnt so far, what needs to be there to ensure patients benefit from the use of genomics in clinical care?
2. What do you see as the future of the genetic counselling profession when genomics is integrated in clinical care?

***Closing***

Is there any other subject or topic you think we need to discuss to better understand your new role?
